# Family and Peer Risk Factors as Predictors of Lifetime Tobacco Use among Iranian Adolescents: Gender Similarities and Differences

**DOI:** 10.5539/gjhs.v6n4p63

**Published:** 2014-04-07

**Authors:** Azam Baheiraei, Farzaneh Soltani, Abbas Ebadi, Mohammad Ali Cheraghi, Abbas Rahimi Foroushani

**Affiliations:** 1 Department of Reproductive Health, Tehran University of Medical Sciences, Tehran, Iran; 2 Community Based Participatory Research Center, Iranian Institute for Reduction of High-Risk Behaviors, Tehran University of Medical Sciences, Tehran, Iran; 3 Behavioral Sciences Research Center (BSRC), Nursing Faculty, Baqiyatallah University of Medical Sciences, Tehran, Iran; 4 School of Nursing and Midwifery, Tehran University of Medical Sciences, Tehran, Iran; 5 Department of Biostatistics, School of Public Health and Institute of Public Health Research, Tehran University of Medical Sciences, Tehran, Iran

**Keywords:** adolescence, tobacco use, risk factors, gender

## Abstract

**Introduction:**

Family and peer risk factors are considered as important predictors of tobacco use in adolescents. Furthermore, information regarding gender differences in lifetime tobacco use of adolescents is essential for designing gender-specific tobacco prevention policies.

**Methods:**

In a cross-sectional population-based study, 870 Iranian adolescents (430 boys and 436 girls) aged 15-18 years old, filled out the adopted form of “Communities That Care Youth Survey”. Four family and two peer risk factors were entered in adjusted logistic regression analyses to predict the lifetime tobacco use (cigarette and smokeless tobacco) in boys and girls, separately.

**Results:**

Boys reported higher prevalence of lifetime cigarettes use compared to girls (22.8% vs. 17.8%, p = 0.04). However, the prevalence of lifetime smokeless tobacco use in girls was the same as boys, even slightly higher (7.9% vs. 7.1%, P=0.5). “Family history of drug use” and “Friends use of drugs” were common risk factors predicting cigarettes and smokeless tobacco use between both genders. On the other hand, other family risk factors included “Poor family management”, “Parental attitude favorable toward drug use” and “Family conflict” were the predictors of lifetime tobacco use only in girls, but not in boys.

**Conclusion:**

Design and implementation of preventative programs for adolescents tobacco use should be conducted with emphasis on the role of smoker parents at home, and friendship with substance user peers with antisocial behaviors. It seems that family risk factors may have more value in prevention of tobacco use in female adolescents.

## 1. Introduction

Despite the global anti-tobacco policies and heightened public awareness of the harmful effects of tobacco, use of tobacco still remains a global public health problem (Kelishadi, 2011). It has been estimated that 5 million deaths occur annually due to smoking, and most of these happen in low and middle income countries (World Health Organization, 2012).

In Iran, over 65% of the population are in the age range of adolescence, which is considered the age of onset of tobacco use (Sarraf-Zadegan, Boshtam & Shahrokhi, 2004; Jafarabadi, Allahverdipour, & Bashirian, 2012). Numerous studies conducted in the country reveal an alarming rate for tobacco and other substances use among Iranian adolescents (Kelishadi et al., 2006; Niknami, Akbari, & Ahmadi, 2008; Rezaei, Nedjat, & Golestan, 2011; Mohammadpoorasl, Nedjat, & Fakhari, 2012; Habib, Shiraz, & Naseri-Kouzehgarani, 2012; Moeini, Poorolajal, & Gharghani, 2012; Baheiraei, Hamzehgardeshi, & Mohammadi, 2013), whereas all the components of the so called MPOWER strategy to control tobacco consumption have begun in Iran and W.H.O (2011) has reported Iran as one of the first 20 countries with the highest achievement in the global fight against tobacco usage. Despite the onset of anti-tobacco policies since the 1990’s in Iran, national studies have documented an alarming rise in adolescents tobacco trends especially among girls (Kelishadi et al., 2006). Results of national studies on tobacco use in Iran indicate reducing prevalence of regular users, but the problem of tobacco use among adolescents remains unresolved (WHO, 2011). For example, it has been reported that 19.4% of Iranian adolescents have smoked in their lifetime, and 28.9% of school pupils occasionally smoke (Jafarabadi et al., 2012). 66.7% of present smokers confess to have experienced their first cigarette at the age of 14 (Moeini et al., 2012), whereas tobacco use in adolescence is considered the gateway to use of drugs such as heroin, cannabis, cocaine, and stimulants, and has a powerful role in prediction of drug-related behaviors and other future problem behaviors.

The most common forms of tobacco use are cigarettes smoking and smokeless tobacco, and compared to users of other forms of tobacco, a large number from both groups use tobacco daily. In spite of reduction in cigarettes use, smokeless tobacco use has recently increased (Centers for Disease Control and Prevention, 2010). It seems that the existence of laws prohibiting smoking in public places and work environment have led the tobacco industries to produce substitutes containing tobacco (Curry, Pederson, & Stryker, 2011). This companies advertise use of smokeless tobacco products as cigarettes substitute in limited availability or when quitting cigarettes (Carpenter, Connolly, & Ayo-Yusuf, 2009), while epidemiological studies have shown a strong positive correlation between use of smokeless tobacco and various cancers such as oropharynx, esophagus, stomach, pancreas, and lung cancers (Lee, 2011).

Effective prevention of tobacco products use in adolescence requires identifying important risk factors in adolescents’ tobacco use initiation. Unlike the developed countries, where adolescents’ drug use predictors have been identified, the above factors have not yet been fully clarified in developing countries (Brook et al., 2006). Many studies have shown that adolescents’ problem behaviors like tobacco use are associated with many factors in various domains, and several psycho-social factors are considered as important variables associated with tobacco use during adolescence (Kokkevi et al., 2007). Among these, family and peer factors are considered important predictors of smoking in adolescents (Patterson, Reid, & Dishion, 1992; Fleming, Kim, & Harachi, 2002; Simons-Morton, Chen, & Abroms, 2004; Tilson, McBride, & Lipkus, 2004).

Many researchers that have studied substance use in adolescents emphasize the social context, in which interactions within the family and quality and quantity of family relationships are considered as key aspects (Dishion, Nelson, & Bullock, 2004). For example, parents tobacco use, quality of parent-children relationship, parental monitoring and clear rules within the family are mentioned (Skinner, Haggerty, & Catalano, 2009). Relationship with deviant or substance-using peers is another vital aspect of social context that could affect adolescents substance use (Barrera et al., 2001; Brook, Brook, & Arencibia-Mireles, 2001). Some researchers believe that having smoker friends is the most important indicator of tobacco use by adolescents (Hoffman, Sussman, & Unger, 2006). Generally, a review of studies conducted in developed countries suggests influence of disrupted families, history of smoking by parents and peers on adolescents substance use (Brook et al., 2006; Kokkevi et al., 2007; Toumbourou, Hemphill, & Tresidder, 2007). Some researchers have gone so far as to consider family and peers as unique factors in substance use (Aseltine, 1995; Beal, Ausiello, & Perrin, 2001; Brook et al., 2001; Bahr, Hoffmann, & Yang, 2005).

Moreover, although some studies have shown predictive impact of gender on substance use in adolescents (Haas, 2004; Brook et al., 2006), some others have shown that the gender difference does not always apply in different societies and cultures (Ledoux, Miller, & Choquet, 2002). Some studies have attributed gender differences to family factors (Rosay, Gottfredson, & Armstrong, 2000; Kelly, Flaherty, & Toumbourou, 2011). For example, they have cited parental monitoring as one of the most important predictors of adolescents’ outcomes (Huebner & Howell, 2003), however the exact nature of these effects has been not fully understood. Some studies have considered parental monitoring more influential in substance use by girls (Choquet, Hassler, & Morin, 2008), whereas in other research, monitoring is more effective among boys (McArdle et al., 2002).

Generally, strong variables regarding experience of tobacco use during adolescence are contravertial. An important part of these controversies is associated with population and cultural diversity in different countries (Barrera et al., 2001; Steptoe, Wardle, & Cui, 2002; Ma, Shive, & Legos, 2003). Research into social and environmental factors associated with increasing substance use in developing countries have less been conducted (Uchtenhagen, 2004). Also, more studies are necessary to expose gender similarities and differences in substance use predictors in social context, especially in different societies and cultures. This issue is extremely important in countries with a young population and limited resources and facilitites. Wheras the above predictors are dissimilar in opposite genders, then prevention programs aiming to affect intended factors will be ineffective. Furthermore, design and implementation of gender-sensitive preventative strategies can be more successful. Thus, the present study has compared predictive risk factors associated with lifetime cigarette and smokeless tobacco use between 15to 18years old Iranian girls and boys, in social context (i.e. family and peer).

## 2. Materials and Methods

### 2.1 Setting and Data Collection

The present article is part of a larger mixed methods study on risk and protective factors associated with adolescents high-risk behaviors. Results of comparison between gender similarities and differences of risk factors associated with lifetime tobacco products use at family and peers levels are presented in this article. The data were collected through a population-based sample in a cross-sectional study. The sample was drawn through a cluster sampling method across Tehran, Iran. In this method, each household was considered as a cluster. First, Tehran was divided into five geographical districts (North, South, East, West, and Center), and sampling was carried out according to neighborhoods located in these five districts, and number of households in each neighborhood. The questionnaire was completed in self-reporting style, supervised by the researcher, in the absence of family members and others. Due to high sensitivity of the issues raised in the questionnaire, verbal consent was obtained from adolescents and their families. Participants were assured of confidentiality of information.

### 2.2 Sample

Participants in this study were 870 Iranian adolescents (430 boys and 436 girls) aged 15-18 years that voluntarily completed the questionnaire in spring 2013. Table 1 summarizes the sample characteristics.

**Table 1:** Participant characteristics (N=870)

	n	%
***Gender***		
Male	430	50.3
Female	436	49.7
***Age***		
15yr	209	24.1
16yr	227	26.2
17yr	203	23.4
18yr	228	26.3
***Family status***		
Living with both parents	774	89.5
Living with mother	41	4.7
Living with father	36	4.2
Living with others	14	1.7
***Mother’s education***		
Under diploma	223	26.5
Diploma	445	52.9
Academic education	173	20.6
***Father’s education***		
Under diploma	202	27.5
Diploma	399	47.2
Academic education	244	28.9
***Mother’s occupational status***		
Employed	221	24.5
Unemployed	648	75.5
***Father’s occupational status***		
Employed	817	93.8
Unemployed	53	6.2
***Lifetime cigarettes smocking use***		
Never	691	79.7
Once or twice	103	11.9
Sometimes but not regularly	49	5.7
Regularly in the past	7	0.8
Regularly now	16	1.8
***Lifetime smokless tobacco use***		
Never	804	92.4
Once or twice	33	3.8
Sometimes but not regularly	27	3.1
Regularly in the past	4	0.5
Regularly now	1	0.1

Note. Sample sizes range (n=841 to n=870) due to item-level missing data

### 2.3 Ethical Consideration

This work was supported by Tehran University of Medical Sciences (grant number 91-03-28-19503). The ethics committee of Tehran University of Medical Science approved the protocol of the study. Participants were assured of confidentiality, and were told they could withdraw whenever they wished.

### 2.4 Measures

The self-reported measures of family and peers risk factors were obtained from a adapted form of the Communities That Care Youth Survey (CTC-YS), which assesses wide ranging risk and protective factors, along with problem behaviors (Arthur et al. 2002). CTC-YS psychometric properties was previously examined by our research team in Iranian adolescents. In a study on 753 Iranian adolescents aged 15-18 years in 2012, reliability of CTC-YS was assessed using Cronbach’s alpha, and its construct validity was assessed using confirmatory factor analysis. The validity and reliability of CTC-YS showed that with slight adjustments, this questionnaire has appropriate psychometric characteristics in Iranian adolescents.

#### 2.4.1 Family Risk Factors

Four scales examined risk factors within the family level:

“Parental attitude favorable toward drug use” was measured through three items such as “In your parents opinions, how wrong is it for you to do the followings: Smoking cigarettes?”

“Family history of drug use” was measured through three items such as “Have any of your brothers or sisters ever: Smoked cigarettes?

“Poor family management” was measured through eight items such as “Would your parents know if you did not come home on time?”

“Family conflict” was measured using two items such as “People in my family have serious arguments.”

#### 2.4.2 Peer Risk Factors

Two scales examined risk factors within the peer level:

“Friends use of drugs” included three items such as “In the past year (12 months), how many of your best friends have smoked cigarettes?”

“Interaction with antisocial peers” included seven items such as “In the past year (12 months), how many of your best friends were suspended or expelled from school?

Each measure was composed of 8-2 questions generally answered on a 3-5 point scale, and α= 0.73-0.81.

#### 2.4.3 Outcome Variables

The Outcome variables included both lifetime use of cigarettes and smokeless tobacco. The outcome was assessed using the following question:

“Have you ever smoked cigarettes/smokeless tobacco?” with responses on a five-point likert scale including “never, once or twice, sometimes but not regularly, regularly in the past and regularly now.”

The outcome variables were recoded to form a dichotomous measure never or no use (0) and responses other than never or none (1).

#### 2.4.4 Control Variables

Control variables included Family status (with or without both parents present in the family), Parental occupational status and Parental education.

### 2.5 Analytic Strategy

Prior to commencement of any statistical analysis, data were suitably arranged for analysis, so that a number of items were reverse-coded again and items with different Likert scales were equalized. Respondents were scored on each risk factor by averaging responses to the items comprising each risk factor and accordingly, they were coded into “low-risk” and “high-risk” (low-risk=1, high-risk=2).

The main statistical analysis included a series of separate logistic regression analyses. Unadjusted logistic regression analyses were performed to examine the potential relationship between each risk factor and lifetime use of cigarette and smokeless tobacco, separately. Then, all rik factors of family and peer levels were entered into the adjusted logistic regression models, respectively. All the analyses for demographic factors had been controlled, and also, all analyses were conducted for girls and boys to predict the lifetime cigarettes and smokeless tobacco use, separately. Other than Family status factor, other demographic factors included Parental education and Parental occupational status did not show any significant correlation with cigarettes or smokeless tobacco use, and therefore they were not entered in the multivariate model. All analyses were conducted using the SPSS19.0 and a *p* value less than 0.05 was considered significant.

## 3. Results

### 3.1 Participants Characteristics

Among the participants, 50.3% were girls and 49.7% boys. 89.5% were high school students, and majority (94.2%) spoke Persian as their first language. Sample characteristics are presented in Table 1. Generally, 20.3% of adolescents reported cigarette smoking experience (17.8% girls, and 22.8% boys), and 7.6% reported use of smokeless tobacco (7.9% girls, and 7.1% boys). Meanwhile, 7.3% of participants were current cigarette users and 4.3% of them were current smokeless tobacco users. Descriptive statistics for each variable are provided in Table 2.

**Table 2:** Particiapnt description

Variable	N	M/Med	SD/Users %
Parental attitude toward drug use (Boys)	436	1.12	0.36
Family history of drug use (Boys)	433	1.70	0.82
Poor family management (Boys)	424	2.38	0.40
Family conflict (Boys)	436	2.19	1.12
Friend use of drugs (Boys)	436	0.75	1.20
Interaction with antisocial peers (Boys)	436	0.46	0.86
Parental attitude toward drug use (Girls)	430	1.19	0.54
Family history of drug use (Girls)	428	1.62	0.74
Poor family management (Girls)	423	2.29	0.43
Family conflict (Girls)	430	2.28	1.14
Friend use of drugs (Girls)	430	0.37	0.57
Interaction with antisocial peers (Girls)	430	0.27	0.47
Cigarettes smoking (Boys)	434	00	22.8
Cigarettes smoking (Girls)	428	00	17.8
Smokeless tobacco (Boys)	436	00	7.1
Smokeless tobacco (Girls)	429	00	7.9

Note. Mean (M) presented for family and peer variables; Median (Med) presented for outcomes. Standard deviation (SD) presented for family and peer variables; percent of users presented for cigarettes and smokeless tobacco use.

### 3.2 Predictors of Lifetime Cigarette use

Table 3 presents lifetime cigarettes use predictors among boys and girls separately. In the unadjusted logistic regression analyses, all risk factors were predictors of lifetime cigarette use in girls, except risk factor of “Interaction with antisocial peers”. The highest OR related to “Parental attitudes favorable toward drug use”. In boys, all risk factors were predictors of lifetime cigarettes use, except “Poor family management”, and the highest OR related to “Interaction with antisocial peers”.

**Table 3:** Unadjusted and adjusted logistic regression analyses examined family and peer risk factors for lifetime cigarettes smoking among boys (n=430) and girls (n= 436)

Variable	Partially adjusted analyses OR (95% CI)		Fully adjusted analyses OR (95% CI)	

	Boys	Girls	Boys	Girls
***Family risk factors***				
Parental attitudes favorable toward drug use	3.58(1.11-11.5) *	4.98(1.77-14.4)***	2.03(0.58-7.02)	3.37 (1.09-10.3) **
Family history of drug use	3.26(1.92-5.55) ***	3.12(1.69-5.74)***	2.83(1.69-4.76)***	2.54(1.35-4.75)***
Poor family management	1.47(0.76-2.84)	3.06(1.43-6.51)***	1.37(0.70-2.65)	2.40(1.13-5.11)**
Family conflict	1.37 (0.85-2.23)	3.02(1.72-5.28)***	1.32(0.80-2.19)	2.65(1.48-4.75)***
***Peer risk factors***				
Friends use of drugs	3.79(1.92-7.48) ***	4.81(1.65-13.9)***	2.54(1.18-5.45)**	4.40(1.49-12.9)***
Interaction with antisocial peers	8.10(2.78-23.5) ***	8.54(.66-109.7)	4.55(1.42-14.5)**	5.54(0.43-70.8)

The partially adjusted analyses control for family status, parent education and parent employment.

The fully adjusted analyses control for family status and all the 6 family and peer risk factors.

OR =Odds Ratio; 95% CI - 95% Confidence Interval.

* p <.05;

** p <.01;

*** p <.001.

By performing adjusted logistic regression analyses, all family risk factors and one of the two peer risk factors named “Friends use of drugs” remained in the girls regression model. With respect to boys, both risk factors associated with the peers level still remained in the regression model, but in the family level, all risk factors were excluded from regression model, except “Family history of drug use”.

Generally, the strongest predictor of lifetime cigarette use in both genders was associated with the peers level, so that in girls, “Friends use of drug” and in boys, “Interaction with antisocial peers” had the strongest predicting ability in lifetime cigarette use. The risk factors of “Family history of drug use” and “Friends use of drugs” were the common predictors of lifetime cigarette use in both genders.

### 3.3 Predictors of Lifetime Smokeless Tobacco Use

Table 4 presents lifetime smokless tobacco use predictors among boys and girls separately. In the unadjusted logistic regression analyses, all risk factors were predictors of lifetime smokeless tobacco use in girls except “Interaction with antisocial peers”. Among these, the highest OR related to “Parental attitudes favorable toward drug use”. On the other hand, in boys two risk factors of “Poor family management” and “Family conflict” did not show any significant relationship with smokeless tobacco use, and the risk factor of “Parental attitudes favorable toward drug use”, showed the highest Odds Ratio.

**Table 4:** Unadjusted and adjusted logistic regression analyses examined peer and family risk factors for lifetime smokeless tobacco among boys (n=430) and girls (n= 436)

Variable	Partially adjusted analyses OR (95% CI)		Fully adjusted analyses OR (95% CI)	

	Boys	Girls	Boys	Girls
***Family risk factors***				
Parental attitudes favorable toward drug use	5.36(1.32-21.7) **	5.74(1.82-18.1) ***	2.98(0.63-13.9)	4.39 (1.26-15.2)***
Family history of drug use	4.25(1.87-9.66)***	4.84(2.23-10.5) ***	3.91(1.69-9.05)***	3.68(1.63-8.27)***
Poor family management	0.72(0.29-1.80)	4.15(1.22-14.1) *	0.75(0.28-1.99)	4.41(1.18-16.4)***
Family conflict	2.10(0.94-4.70)	2.88(1.32-6.26) ***	1.83(0.78-4.25)	2.42(1.06-5.52)***
***Peer risk factors***				
Friends use of drugs	4.67(1.84-11.86)***	5.21(1.59-17.02)***	3.67(1.27-10.5)***	4.90(1.49-16.12)***
Interaction with antisocial peers	5.11(1.46-17.84)*	4.74(0.34-65.26)	2.23(0.53-9.40)	3.27(0.24-43.48)

The partially adjusted analyses control for family status, parent education and parent employment.

The fully adjusted analyses control for family status and all the 6 family and peer risk factors.

OR =Odds Ratio; 95% CI - 95% Confidence Interval.

* p <.05;

** p <.01;

*** p <.001.

By performing adjusted logistic regression analyses, still all family level risk factors and one peers level risk factor (Friends use of drugs), remained in the girls regression model. With respect to boys, only one risk factor from family level (Family history of drug use) and one risk factor from peers level (Friends use of drugs) remained in the regression model. “Friends use of drugs” and “Family history of drug use” were common risk factors predicting smokeless tobacco use between both genders.

## 4. Discussion

Problem behaviors like tobacco, alcohol and other substances use, mental problems, unsafe sex, unsafe driving and violence that increase adolescents short and long-terms mortality and morbidity, are largely preventable (Catalano, Fagan, & Gavin, 2012). Meanwhile, lack of attention to gender differences has created a large gap in the prevention of problem behaviors (Fagan, Van Horn, & Hawkins, 2007).

Although male gender is the biggest predictor of worldwide tobacco use with prevalence of 4 to 1 compared to women (Corrao, Guindon, & Ckkinides, 2000), the joint W.H.O and C.D.C study of gender differences in tobacco use in the six regions worldwide was indicative of reduced gender differences in cigarette use and especially other tobacco products use between girls and boys, so that, in 70.1% users of other tobacco products (other than cigarette), no gender difference was observed (Global Youth Tobacco Survey Collaborating Group, 2003). Difference Reduction in prevalence of cigarette smoking between girls and boys, and also lifetime use of smokeless tobacco by girls, every step with boys, should draw our attention to the warning of the World Health Organization in the introduction to tobacco control treaty about the increase in smoking cigarettes and other forms of tobacco consumption by women and young girls worldwide (Samet & Yoon, 2010).

In the present study, boys had higher prevalence of lifetime cigarettes use compared to girls. However, the prevalence of lifetime smokeless tobacco use in girls was the same as boys, even slightly higher. Other researchers also found that gender differences in use of substances are decreasing (Sanchez, Opaleye, & Martins, 2010). Some researchers argue that the reason for this decreasing difference is the girls’ tendency to compete with older boys, who are more likely to use substances because of their age group (Johnston, O’Malley, & Bachman, 2012). Pinilla, González and Barber (2002) debated this worrying hypothesis that in perception of girls, being male and powerful, has increased tobacco smoking attraction among them.

Gender differences associated with substance use may be due to socio-cultural differences in terms of available opportunities for adolescents (Caetano, Clark, & Tam, 1998; Wichstrøm, 2001), or these differences may be associated with boys and girls tobacco type preferences (Grunberg, Winders, & Wewers, 1991). It seems that both these factors are somehow implied in the present study, so that, adolescent girls have a higher tendency toward use of other tobacco products, like smokeless tobacco, or hookah, due to cigarette smoking being considered unbecoming for girls and women in Iranian culture. For example, common use of hookah by girls and women in Iranian society can be cited (Kelishadi et al., 2007). As the World Health Organization emphasizes, further studies should be conducted about women’s use of different type of tobacco products such as snuff, chewing tobacco, smokeless tobacco, etc, especially in the developing countries (Samet & Yoon, 2010).

Principle aim of the present study has been to compare Iranian adolescent boys and girls in terms of predictors of lifetime use of tobacco products in social context (i.e. family and peers). In the present study, the common predicting factor in both gender, in cigarette use as well as smokeless tobacco use, was “Family history of drug use”, which was a prticularly strong predictor among family factors in boys. In fact, this was the only family risk factor that remained in the boys regression model after performing multivariate regression analyses. The issue that use of cigarettes and other tobacco products is higher in adolescents with drug user parents, has been ascertained by researchers for years (Karimy, Niknami, & Heidarnia, 2013; Flay, Hu, & Richardson, 1998; Tilson et al., 2004; Hill, Hawkins, & Catalano, 2005; Brook et al., 2006; Skinner et al., 2009; Baheiraei et al., 2013). In a study conducted in Tehran in relation to tobacco use indicators in uptown and downtown areas of the city, the only common predictor of cigarette use in both socio-economic areas was having a smoker father (Rezaei et al., 2011).

Although, in many cases, it may be that parents substance use per se is not necessarily a risk factor, rather, the parenting style and family environment resulting from substance user parents may make a greater impact (Swadi, 1994). In the present study, this issue applied more to girls than to boys, so that in addition to the “Family history of drug use” risk factor, other family risk factors such as “Poor family management”, and “Parental attitude favorable toward drug use” with high OR as well as “Family conflict” were the predictors of lifetime tobacco product use only in girls, but not in boys. It is argued that girls are probably more sensitive to family issues, or since they spend more time indoors compared to boys, they interact more with parents and are more exposed to family conflicts (Fagan et al., 2007), As resarchers have shown that the most effective factor in family interactions is length of time spent with the family (Adlaf & Ivis, 1997). However our study is in agreement with studies that found family factors stronger predictors in girls than boys (Farrington & Painter, 2004; Blitstein, Murray, & Lytle, 2005; Yeh, Chiang, & Huang, 2006; Choquet et al., 2008; Sanchez, Opaleye, & Martins, 2010; Kelly et al., 2011), Other studies have found the above factors more dominant in boys (Moffitt, Caspi, & Rutter, 2001; Piquero & Sealock, 2004) or have not found a significant difference between boys and girls in terms of existing risk factors in the family (Loeber, & Stouthamer-Loeber, 1986; Rowe, Flannery, & Flannery, 1995; Fergusson & Horwood, 2002). However, there are very few longitudinal studies about gender differencs.

In the present study, despite family factors being more significant in girls compared to boys, the highest Odds Ratio (especially in girls) related to the peer level’s predicting factors. Furthermore, risk factors of both family and peer levels combined together, were observed more in girls, which probably indicates the interaction between family and peer risk factors to push girls toward use of tobacco products. As resarchers have shown, parents that neglect their duties and active involvement and monitoring children, can lay the grounds for influence of antisocial friends (Van Ryzin, Fosco, & Dishion, 2012). Parents’ influence may be through their assertion in choosing youth friends (Engels, Vitaro, & Blokland, 2004), which in turn influences adolescent attitudes toward tobacco use. Most studies have shown that this impact is the same in boys and girls (Urberg, Değirmencioğlu, & Pilgrim, 1997; Kokkevi et al., 2007; García-Rodríguez, Suárez-Vázquez, & Secades-Villa, 2010), but our study indicated highly important impact of family attributes on girls behaviors more than boys. It appears family, that is the most important, most noble, and decisive institution in producing children’s personality basis in Iran, has a particular sensitivity in girls. Studies based on evolutionary theories have shown that perceptions and reactions of girls may be different from boys toward family factors, and consequently they are not equally exposed to risks (Pilgrim, Schulenberg, & O’Malley, 2006). However, studies on differences in mechanism of perception and experience of girls and boys of risk and protection factors have not yet been conclusive (Fagan et al., 2007).

Advantages of the present study include being population-based, equal number of boys and girls in the study, and assessing prevalence of lifetime use of smokeless tobacco and its social risk factors alongside lifetime cigarettes use in Iranian adolescents, which had not been considered in other studies.

The main limitation in the present study is its cross-sectional nature, which limits the possibility of establishing a causal relationship between variables. Conducting longitudinal, or even experimental studies to investigate the relationship between family and peer risk factors, and tobacco use among Iranian adolescents age and gender groups is required. The present study only considered family and peers risk factors, and the decisive roles of protective factors in these levels, singularly or in interaction with risk factors, in adolescent tobacco use have not been considered. Furthermore, other risk and protective factors in the individual, school, and community levels, each singularly, or in interaction with others can push adolescents toward tobacco products use, or conversely, prevent them from it.

## 5. Conclusion

Given the common risk factors between adolescent boys and girls in the present study, design and implementation of preventative programs for adolescents tobacco use should be conducted with emphasis on the role of smoker parents at home, and friendship with substance user peers with antisocial behaviors, is also highly imortant in both genders (especially in boys). The influence of family factors on girls lifetime cigarettes and smokeless tobacco use was found to be higher than on boys in the present study. Combined family and peer risk factors together to predict lifetime use of tobacco products can be an example of interaction between these two groups of factors to push girls toward substance use, which needs to be confirmed through further and more in-depth studies.

The above gender differences in social predictors of lifetime tobacco use is a key factor in future preventative programs on adolescents and provides important perspectives in influential factors of tobacco use in girls and boys. These data can be used in the design of preventive elective strategies, especially in countries with limited resources.

## References

[ref1] Adlaf E. M, Ivis F. J (1997). Structure and Relations: The Influence of Familial Factors on Adolescents Substance Use and Delinquency. Journal of Child & Adolescent Substance Abuse.

[ref2] Arthur M. W, Hawkins J. D, Pollard J. A (2002). Measuring risk and protective factors for substance use, delinquency, and other adolescent problem behaviors: The Communities That Care Youth Survey. Evaluation Review.

[ref3] Aseltine J. R (1995). A reconsideration of parental and peer influences on adolescent deviance. Journal of health and social behavior.

[ref4] Baheiraei A, Hamzehgardeshi Z, Mohammadi M. R (2013). Personal and Family Factors Affecting Life time Cigarette Smoking among Adolescents in Tehran (Iran): A Community Based Study. Oman medical journal.

[ref5] Bahr S. J, Hoffmann J. P, Yang X (2005). Parental and peer influences on the risk of adolescent drug use. Journal of Primary Prevention.

[ref6] Barrera M, Biglan A, Ary D (2001). Replication of a problem behavior model with American Indian, Hispanic, and Caucasian youth. Journal of Early Adolescence.

[ref7] Beal A. C, Ausiello J, Perrin J. M (2001). Social influences on health-risk behaviors among minority middle school students. Journal of Adolescent Health.

[ref8] Blitstein J. L, Murray D. M, Lytle L. A (2005). Predictors of violent behavior in an early adolescent cohort: Similarities and differences across genders. Health Education & Behavior.

[ref9] Brook J. S, Brook D. W, Arencibia-Mireles O (2001). Risk factors for adolescent marijuana use across cultures and across time. The Journal of genetic psychology.

[ref10] Brook J. S, Morojele N. K, Pahl K (2006). Predictors of drug use among South African adolescents. Journal of Adolescent Health.

[ref11] Centers for Disease Control and Prevention (2010). Vital Signs: Current Cigarette Smoking Among Adults Aged 18 Years—United States 2009.

[ref12] Caetano R, Clark C. L, Tam T (1998). Alcohol consumption among racial/ethnic minorities. Alcohol health and research world.

[ref13] Canter R. J (1982). Family correlates of male and female delinquency. Criminology.

[ref14] Carpenter C. M, Connolly G. N, Ayo-Yusuf O. A (2009). Developing smokeless tobacco products for smokers: an examination of tobacco industry documents. Tobacco Control.

[ref15] Catalano R. F, Fagan A. A, Gavin L. E (2012). Worldwide application of prevention science in adolescent health. The Lancet.

[ref16] Choquet M, Hassler C, Morin D (2008). Perceived parenting styles and tobacco, alcohol and cannabis use among French adolescents: Gender and family structure differentials. Alcohol and Alcoholism.

[ref17] Corrao M. A, Guindon G. E, Cokkinides V (2000). Building the evidence base for global tobacco control. Bulletin of the World Health Organization.

[ref18] Curry L. E, Pederson L. L, Stryker J. E (2011). The changing marketing of smokeless tobacco in magazine advertisements. Nicotine & Tobacco Research.

[ref19] Dishion T. J, Nelson S. E, Bullock B. M (2004). Premature adolescent autonomy: Parent disengagement and deviant peer process in the amplification of problem behavior. Journal of Adolescence.

[ref20] Engels R. C, Vitaro F, Blokland E. D (2004). Influence and selection processes in friendships and adolescent smoking behaviour: the role of parental smoking. Journal of Adolescence.

[ref21] Fagan A. A, Van Horn M. L, Hawkins J. D (2007). Gender similarities and differences in the association between risk and protective factors and self-reported serious delinquency. Prevention Science.

[ref22] Farrington D. P, Painter K. A (2004). Gender differences in offending: Implications for risk-focused prevention, Home Office London.

[ref23] Fergusson D. M, Horwood L. J (2002). Male and female offending trajectories. Development and psychopathology.

[ref24] Flay B. R, Hu F. B, Richardson J (1998). Psychosocial predictors of different stages of cigarette smoking among high school students. Preventive medicine.

[ref25] Fleming C. B, Kim H, Harachi T. W (2002). Family processes for children in early elementary school as predictors of smoking initiation. Journal of Adolescent Health.

[ref26] Mohammadpoorasl A, Nedjat S, Fakhari A (2012). Smoking Stages in an Iranian Adolescent Population. Acta Medica Iranica.

[ref27] García-Rodríguez O, Suárez-Vázquez R, Secades-Villa R (2010). Smoking risk factors and gender differences among Spanish high school students. Journal of drug education.

[ref28] Global Youth Tobacco Survey Collaborating Group (2003). Differences in worldwide tobacco use by gender: findings from the Global Youth Tobacco Survey. Journal of School Health.

[ref29] Grunberg N. E, Winders S. E, Wewers M. E (1991). Gender differences in tobacco use. Health Psychology.

[ref30] Haas K (2004). Relationship of gender to licit and illicit drug use among adolescents. Annual Review of Undergraduate Research at the College of Charleston.

[ref31] Habib E, Shiraz A. S, Naseri-Kouzehgarani G (2012). The determinants of high school students smoking habits with special focus on teachers smoking in Iran: a population based study. Pneumologia (Bucharest, Romania).

[ref32] Hill K. G, Hawkins J. D, Catalano R. F (2005). Family influences on the risk of daily smoking initiation. Journal of Adolescent Health.

[ref33] Hoffman B. R, Sussman S, Unger J. B (2006). Peer influences on adolescent cigarette smoking: A theoretical review of the literature. Substance use & misuse.

[ref34] Huebner A. J, Howell L. W (2003). Examining the relationship between adolescent sexual risk-taking and perceptions of monitoring, communication, and parenting styles. Journal of Adolescent Health.

[ref35] Jafarabadi M. A, Allahverdipour H, Bashirian S (2012). Modeling the Underlying Predicting Factors of Tobacco Smoking among Adolescents. Iranian journal of public health.

[ref36] Johnston L. D, O’Malley P. M, Bachman J. G, Schulenberg J. E (2012). Monitoring the Future national results on adolescent drug use: Overview of key findings 2011. Ann Arbor: Institute for Social Research.

[ref37] Karimy M, Niknami S, Heidarnia A. R (2013). Prevalence and Determinants of Male Adolescents’ Smoking in Iran: An Explanation Based on the Theory of Planned Behavior. Iranian Red Crescent Medical Journal.

[ref38] Kelishadi R (2011). Tobacco use prevention for Iranian adolescents: Time for family-centered counseling programs. International journal of preventive medicine.

[ref39] Kelishadi R, Ardalan G, Gheiratmand R, Majdzadeh R, Delavari A, Heshmat R, Barekati H (2006). Smoking behavior and its influencing factors in a national-representative sample of Iranian adolescents: CASPIAN study. Preventive medicine.

[ref40] Kelishadi R, Mokhtari M, Tavasoli AA, Khosravi A, Ahangar-Nazari I, Sabet B, Amini A (2007). Determinants of tobacco use among youths in Isfahan, Iran. International Journal of Public Health.

[ref41] Kelly A. B, Flaherty M, Toumbourou J. W (2011). Gender differences in the impact of families on alcohol use: a lagged longitudinal study of early adolescents. Addiction.

[ref42] Kelly A. B, Toumbourou J. W, O’Flaherty M (2011). Family relationship quality and early alcohol use: evidence for gender-specific risk processes. Journal of studies on alcohol and drugs.

[ref43] Kokkevi A, Richardson C, Florescu S (2007). Psychosocial correlates of substance use in adolescence: A cross-national study in six European countries. Drug and Alcohol Dependence.

[ref44] Kokkevi A. E, Arapaki A. A, Richardson C (2007). Further investigation of psychological and environmental correlates of substance use in adolescence in six European countries. Drug and Alcohol Dependence.

[ref45] Ledoux S, Miller P, Choquet M (2002). Family structure, parent–child relationships, and alcohol and other drug use among teenagers in France and the United Kingdom. Alcohol and Alcoholism.

[ref46] Lee P. N (2011). Summary of the epidemiological evidence relating snus to health. Regulatory Toxicology and Pharmacology.

[ref47] Loeber R, Stouthamer-Loeber M (1986). Family factors as correlates and predictors of juvenile conduct problems and delinquency. Crime & Just.

[ref48] Ma G. X, Shive S, Legos P (2003). Ethnic differences in adolescent smoking behaviors, sources of tobacco, knowledge and attitudes toward restriction policies. Addictive Behaviors.

[ref49] McArdle P. A, Wiegersma A, Gilvarry E, Kolte B, McCarthy S, Fitzgerald M, Quensel S (2002). European adolescent substance use: the roles of family structure, function and gender. Addiction.

[ref50] Moeini B, Poorolajal J, Gharghani Z. G (2012). Prevalence of Cigarette Smoking and Associated Risk Factors among Adolescents in Hamadan City, West of Iran in 2010. Journal of Research in Health Sciences.

[ref51] Moffitt T. E, Caspi A, Rutter M (2001). Sex differences in antisocial behaviour: Conduct disorder, delinquency, and violence in the Dunedin Longitudinal Study.

[ref52] Niknami S, Akbari M, Ahmadi F (2008). Smoking initiation among Iranian adolescents: a qualitative study. East Mediterr Health Journal.

[ref53] Patterson G. R, Reid J. B, Dishion T. J (1992). Antisocial boys.

[ref54] Pilgrim C. C, Schulenberg J. E, O’Malley P. M (2006). Mediators and moderators of parental involvement on substance use: A national study of adolescents. Prevention Science.

[ref55] Pinilla J, González B, Barber P (2002). Smoking in young adolescents: an approach with multilevel discrete choice models. Journal of epidemiology and community health.

[ref56] Piquero N. L, Sealock M. D (2004). Gender and general strain theory: A preliminary test of Broidy and Agnew’s gender/GST hypotheses. Justice Quarterly.

[ref57] Rezaei F, Nedjat S, Golestan B (2011). Comparison of Onset Age and Pattern of Male Adolescent Smoking in Two Different Socioeconomic Districts of Tehran, Iran. International journal of preventive medicine.

[ref58] Rosay A. B, Gottfredson D. C, Armstrong T. A Invariance of measures of prevention program effectiveness: A replication. Journal of Quantitative Criminology.

[ref59] Rowe D. C, Flannery A. T, Flannery D. J (1995). Sex differences in crime: Do means and within-sex variation have similar causes?. Journal of Research in Crime and Delinquency.

[ref60] Samet J. M, Yoon S. Y (2010). Gender, women and the tobacco epidemic. World Health Organization.

[ref61] Sanchez Z, Opaleye E, Martins S (2010). Adolescent gender differences in the determinants of tobacco smoking: a cross sectional survey among high school students in São Paulo. BMC public health.

[ref62] Sarraf-Zadegan N, Boshtam M, Shahrokhi S (2004). Tobacco use among Iranian men, women and adolescents. The European Journal of Public Health.

[ref63] Simons-Morton B, Chen R, Abroms L (2004). Latent growth curve analyses of peer and parent influences on smoking progression among early adolescents. Health Psychology.

[ref64] Skinner M. L, Haggerty K. P, Catalano R. F (2009). Parental and peer influences on teen smoking: Are White and Black families different?. Nicotine & Tobacco Research.

[ref65] Steptoe A, Wardle J, Cui W (2002). An international comparison of tobacco smoking, beliefs and risk awareness in university students from 23 countries. Addiction.

[ref66] Swadi H (1994). Parenting capacity and substance misuse: an assessment scheme. ACPP Review and Newsletter.

[ref67] Tilson E. C, McBride C. M, Lipkus I. M (2004). Testing the interaction between parent–child relationship factors and parent smoking to predict youth smoking. Journal of Adolescent Health.

[ref68] Toumbourou J. W, Hemphill S. A, Tresidder J (2007). Mental health promotion and socio-economic disadvantage: lessons from substance abuse, violence and crime prevention and child health. Health promotion journal of Australia.

[ref69] Uchtenhagen A (2004). Substance use problems in developing countries. Bulletin of the World Health Organization.

[ref70] Urberg K. A, Değirmencioğlu S. M, Pilgrim C (1997). Close friend and group influence on adolescent cigarette smoking and alcohol use. Developmental psychology.

[ref71] Van Ryzin M. J, Fosco G. M, Dishion T. J (2012). Family and peer predictors of substance use from early adolescence to early adulthood: An 11-year prospective analysis. Addictive Behaviors.

[ref72] World Health Organization (2011). WHO Report on the global tobacco epidemic: Warning about the dangers of tobacco.

[ref73] World Health Organization (2012). WHO Report on the global tobacco epidemic 2009: Implementing smoke-free environments.

[ref74] Wichstrøm L (2001). The impact of pubertal timing on adolescents’ alcohol use. Journal of Research on Adolescence.

[ref75] Yeh M. Y, Chiang I. C, Huang S. Y (2006). Gender differences in predictors of drinking behavior in adolescents. Addictive Behaviors.

